# Study of the pathogenic potential of *Dientamoeba fragilis* in experimentally infected mice

**DOI:** 10.1016/j.parepi.2016.05.002

**Published:** 2016-05-28

**Authors:** Eman K. El-Gayar, Amira B. Mokhtar, Wael A. Hassan

**Affiliations:** a Medical Parasitology Department, Faculty of Medicine, Suez Canal University, Egypt; b Pathology Department, Faculty of Medicine, Suez Canal University, Egypt, Ismailia, 41522, Egypt

**Keywords:** *Dientamoeba fragilis*, Experimental mice infection, Infective dose

## Abstract

*Dientamoebafragilis* (*D. fragilis*) is a protozoan parasite whose pathogenic potential is still disputable. The aim of this study was to illustrate the pathogenicity of *D. fragilis* infection and to determine the infective dose for experimental mice infection. Three groups of mice (8/each) were orally inoculated with *in vitro* cultured *D. fragilis*. The infected groups (G1- G3) received 10^3^, 10^5^ and 4 × 10^6^*D. fragilis*/0.5 ml culture, respectively. A control group (G4) only received parasite-free culture. Two weeks post-inoculation all mice were euthanized for histopathological examination. All mice of G3 (100%) and three mice of G2 (37.5%) were infected, and the results were confirmed by PCR and different staining methods. On the other hand, all mice from group G1 showed a completely negative result. Histopathological examination of the colon and caecum of the highly infected group G3 showed active colitis, with infiltration of mixed inflammatory cells such as eosinophils, neutrophils and lymphocytes within the lamina propria of the intestinal wall. The parasite was not invading the colonic mucosa. This study revealed that infection with *D. fragilis* is dose-dependent. Moreover, a dose of 10^5^*D. fragilis*/mouse or higher is necessary to infect mice through the oral route. In addition, this route of infection, although non-invasive, can induce severe inflammatory changes to the colonic and caecal mucosa in experimentally infected mice.

## 1 Introduction

*Dientamoeba fragilis* was first described in 1918 as a binucleated, unflagellated protozoan that inhabits the large intestine (Jepps and Dobell, 1918). On electron microscopy, it has been reclassified as an amoeba‐flagellate rather than an amoeba (Camp et al., 1974). It has a worldwide distribution in both urban and rural areas with infection rates ranging from 0.5% to 16%, where higher rates were reported in outbreaks and associated to the lack of personal hygiene (Girginkardeşler et al., 2008). In adults, asymptomatic colonization is present in 75‐85% of individuals affected by the parasite while in children; disease develops in as many as 90% of those colonized (Crotti et al., 2005). Similar to some other parasites (*e.g.*, *Giardia lamblia*, *Cyclospora cayetanensis*, *Cryptosporidium spp.*), the parasite *D. fragilis* has been showed to cause disease in humans regardless of their immune status. Abdominal pain/discomfort and diarrhea are the symptoms most often described in patients harboring *Dientamoeba*. Other symptoms may include fecal urgency, vomiting, nausea, anorexia, weight loss and fever (Cuffari et al., 1998, Norberg et al., 2003, Stark et al., 2010). The parasite is increasingly recognized as a relatively common cause of human diarrhea and long-term chronic infections is commonly observed in infected patients (Stark et al., 2010). *Dientamoeba fragilis* is often associated with other intestinal parasites. A study investigated 1497 confirmed *D. fragilis* cases and found coinfections with *Blastocystis* spp. in 40.3%, with *Endolimax nana* in 24%, and with *Entamoeba coli*, *G. lamblia* and *Entrobius vermicularis* in 6, 5.7 and 5% of the cases, respectively (Ayadi and Bahri, 1999).

*D. fragilis* cells tend to infect the mucosal crypts of the large intestine from the caecum to the rectum, which is located close to the mucosal epithelium (Levine et al., 1980). In addition, this parasite is not known to be invasive and does not cause cellular damage. It may elicit an eosinophilic inflammatory response in the colonic mucosa. Thus, symptoms are related to the irritation of the superficial colonic mucosal, and it has been reported to be associated with marked peripheral eosinophilia (Gray et al., 2013).

Despite the evidence supporting its pathogenic nature (Stark et al., 2006), it is apparent that a degree of uncertainty still surrounds the pathogenic potential of *D. fragilis* (Ayadi and Bahri, 1999, Banik et al., 2011). To be recognized as a true pathogen Koch's postulate must be fulfilled for *Dientamoeba* as a cause of gastrointestinal illness. Studies conducted to investigate the biology of this parasite are limited by methods of *in vitro* cultivation, with difficulties in establishment of long-term cultures. It is commonly observed that *D. fragilis* grows *in vitro* for only few subcultures before dying out (Clark and Diamond, 2002, Munasinghe et al., 2012). Moreover, the lack of an animal model for dientamoebiasis hinders the ability to demonstrate its pathogenicity (Barratt et al., 2011). However, recently few experimental studies have been carried out (Munasinghe et al., 2013, Eida et al., 2015). This study aimed to investigate the suitability of mice as an animal model for experimental *D. fragilis* infection, and to determine the required infective dose. In addition, we studied the pathological changes caused by *D. fragilis* infection in experimentally-challenged mice.

## 2 Subjects and methods

### 2.1 Isolation of *D. fragilis*

The study was conducted from September 2015 to February 2016, in the Parasitology Department, Faculty of Medicine, Suez Canal University, Ismailia. Fecal specimens were collected from patients seeking medical care for different gastrointestinal complaints such as acute or chronic intermittent diarrhea, or diarrhea alternating with constipation, with or without abdominal pain and attending Outpatient Clinics of Suez Canal University and General Hospitals (Ismailia, Egypt). Fresh stool specimens were immediately examined microscopically by direct smear and Lugol's iodine for detection of *D. fragilis* and to exclude other intestinal parasites followed by formalin ethyl acetate concentration technique (Garcia, 2009). Positive samples for *D. fragilis* were further stained with 10% Giemsa stain (Crotti et al., 2005). Modified acid-fast and trichrome stains were performed to exclude infection of patients with other parasites *e.g. Cryptosporidium*, *Cyclospora*, *Cystoisospora* and *Microsporidium* spp. (Garcia, 2009). Culture for common enteric bacterial pathogens was done to exclude them (LiPuma et al., 2007). Culture on Jones' media without rice starch was done to exclude infection with *Blastocystis* spp. Positive samples for *D. fragilis* were cultivated in the modified Boeck and Drbohlav (MBD) medium supplemented with antibiotics (Sawangjaroen et al., 1993). The culture was incubated at 37°C for 96h. Each day, the sediment of the culture tubes was examined by light microscopy with × 40 objectives for trophozoites. Daily count of the number of *D. fragilis* trophozoites was done using a Neubauer chamber to adjust the inoculation dose to 10^3^, 10^5^ and 4 × 10^6^ *D. fragilis*/0.5 ml culture medium.

### 2.2 Experimental animal infection

Throughout the study, thirty-two 5–6weeks old immune-competent albino Balb/c mice, weighted 25–30 g, were purchased from the Veterinary Medicine Animal Lab, Suez Canal University. Mice were housed independently in good ventilated, filter-top cages and provided sterile rodent chow and water *ad libitum*. The cage bedding was changed every day to avoid and reduce the potential for fecal contamination occurring during the experiment. The animals were maintained in animal house at the faculty of Medicine, Suez Canal University at 25°C, and with a relative humidity of 40–60%.The mice were confirmed to be parasite-free by screening for several days by light microscopy, modified acid-fast and trichrome stain fecal smears for protozoa prior to infection with *D. fragilis*. Mice were randomly divided into four groups. The first three groups were inoculated with *D. fragilis* harvested from 4day-old cultures, at different doses: 10^3^, 10^5^, and 4×10^6^ *D. fragilis* trophozoites/0.5 ml culture medium given orally to G1, G2 and G3, respectively. The fourth group G4 (uninfected control) was given parasite-free culture media (0.5 ml/mouse). Oral inoculation was performed *via* 18G ball-tipped feeding needle attached to 1 ml syringe. All mice were monitored daily for weight loss, presence of loose stool or mucous in stool, lethargy and fur loss from day 1 to day 14 post inoculation. All mice were euthanized 2weeks post-inoculation. All applicable international, national, and/or institutional guidelines for the care and use of animals were followed in this study.

### 2.3 Detection of *D. fragilis* in feces and intestine

Feces from all mice were examined microscopically by wet mount preparation at the 2nd, 4th and 8th day post-inoculation; also the intestinal content of sacrificed mice was examined. Culture on MBD media was done for negative specimens. In this case, cultures were considered negative if the organism was absent until the 7th day post-challenge.

To confirm the presence of *D. fragilis* in infected mice, genomic DNA was extracted from feces of all mice in the infected groups (G1–G3) using the QiaAMP DNA Stool Mini Kit (Qiagen, Hilden, Germany) according to the manufacturer's instructions. DNA was kept frozen at − 20°C until PCR was performed. Then, conventional PCR targeting the SSUrRNA of *D. fragilis* was performed using the following primers; DF400 (5′TATCGGAGGTGGTAATGACC3 ′) and DF1250 (5′CATCTTCCTCCTGCTTAGACG3 ′). The reaction conditions were as follows: pre-denaturation at 94°C for 3 min, followed by 30 cycles of denaturation at 94°C for 1 min, annealing at 57°C for 1.5min, and extension at 72°C for 2min. DNA extracted from a *D. fragilis* positive sample was used as a positive control for PCR. A negative control PCR reaction mixture containing no input DNA was used. The amplified products were separated by electrophoresis in 1% agarose gels (Sigma, USA) in Tris–borate–EDTA buffer. Gels were stained with 0.5% ethidium bromide and photographed using an ultraviolet gel documentation system (UVP Biospectrum, UK) for the presence of specific bands at 870bp (Stark et al., 2005).

### 2.4 Histopathological examination

Two weeks post infection mice were anesthetized and euthanized. Large intestine (colon and caecum) was assessed by naked eye, removed, preserved in 10% formalin, cut into three to five equal cross-sections and processed in paraffin blocks. Intestinal sections (4–8μm thickness) were stained with hematoxylin and eosin (H&E) for histopathological examination. Three sections were made from each tissue specimen (each separated by at least 500μm). Histopathological changes were scored blindly for each mouse. Degree of inflammation was scored from 0 to 4 (0, normal; 1, mucosal hyperplasia; 2, spotty infiltration by inflammatory cells not involving the entire thickness of the mucosa; 3, marked increase of inflammatory cells involving full thickness of mucosa; 4, marked increase of inflammatory cells in mucosa and submucosa, with tissue intact architecture (Hamano et al., 2006).

## 3 Results

### 3.1 Infectivity of *D. fragilis*

The results obtained from mice inoculated orally with *D. fragilis* are summarized in Table 1. During the period of the experiment, no infection occurred in the first group (G1), three mice became infected in G2 and the infection appeared on the fourth day post-inoculation, while all mice in G3 became infected 2 days post-inoculation. All mice in G3 showed loss of weight 2.5–3 g (about10% of the original body weight), decreased activity and diarrheic loose stool. While the three infected mice in G2 showed semi-formed stool without loss of weight.

**Table 1:** infectivity of *D. fragilis* to study groups through oral experimental infection.

Studied group	Inoculum^a^	N of mice/+ (%)
G1	10^3^	8/0 (0)
G2	10^5^	8/3 (37.5)
G3	4 × 10^6^	8/8 (100)

a Number of *D. fragilis* in 0.5 ml culture media.

### 3.2 Detection of *D. fragilis* in stools and intestinal contents

**Fig. 1:**
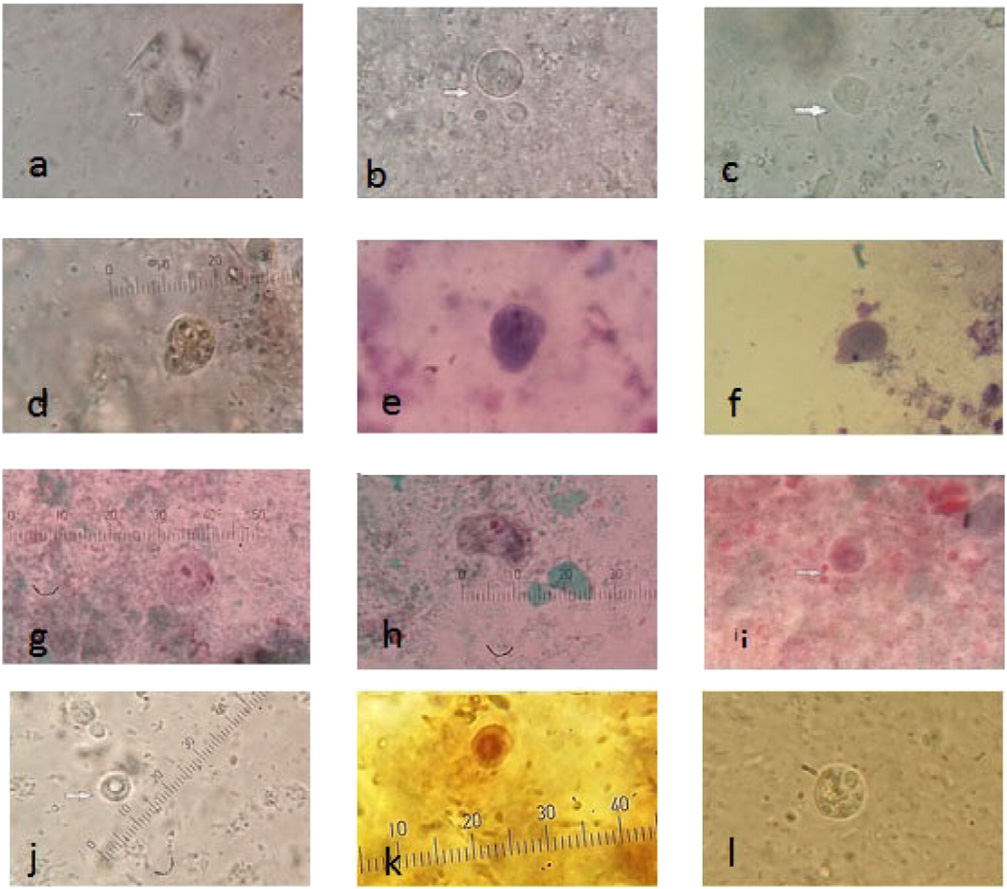
*D. fragilis* trophozoite irregular and rounded forms in wet mount (a – c), irregular form showing inclusions of rice (d), Giemsa-stained trophozoites binucleated (e) and uninucleated (f), trichrome-stained trophozoite (g, h) and precyst (i), presumptive cyst by wet mount ( j) and iodine-stained (k), rounded form trophozoite in MBD culture.

**Fig. 2:**
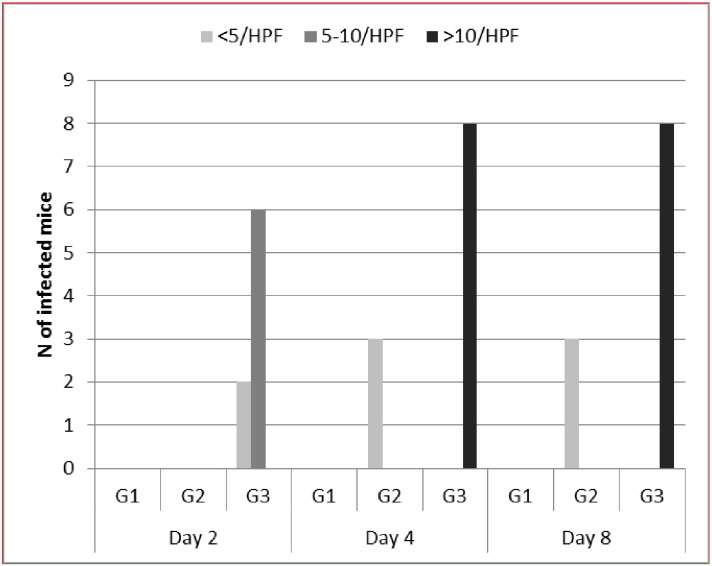
*Dientamoeba fragilis* intensity in direct stool smears of infected mice at different intervals post-inoculation. Data are expressed as average N/HPF.

**Fig. 3:**
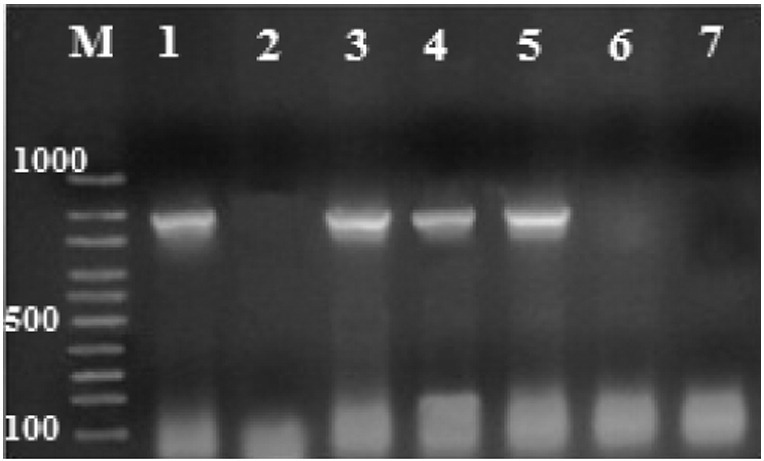
PCR products of *D. fragilis* isolated from stool samples of infected mice on 1% agarose gel stained with ethidium bromide. Lane M, molecular weight marker (100bp); lane 1, positive control; lane 2, negative control; lanes 3–5, infected animal samples with positive amplification of *D. fragilis* SSU rRNA at 870bp. 6 and 7 correspond to negative animal samples.

By direct examination of stool samples both trophozoite and presumptive cyst stages were detected in infected mice (Fig. 1).The average number of *D. fragilis* (trophozoite & presumptive cyst forms)/high power field (HPF) in the stool of the infected mice in each of the infected groups is shown in Fig. 2. Culture in MBD media was performed for positive stool samples to confirm the presence of the parasite. The stool samples and intestinal contents of all mice in G1 and the five mice in G2 which were microscopically negative were cultivated in MBD media and examined for 7days but no growth was detected. All infected mice with *D. fragilis* were confirmed by PCR through detection of the 870bp band targeting the SSUrRNA (Fig. 3).

### 3.3 Histolopathological examination

**Fig. 4:**
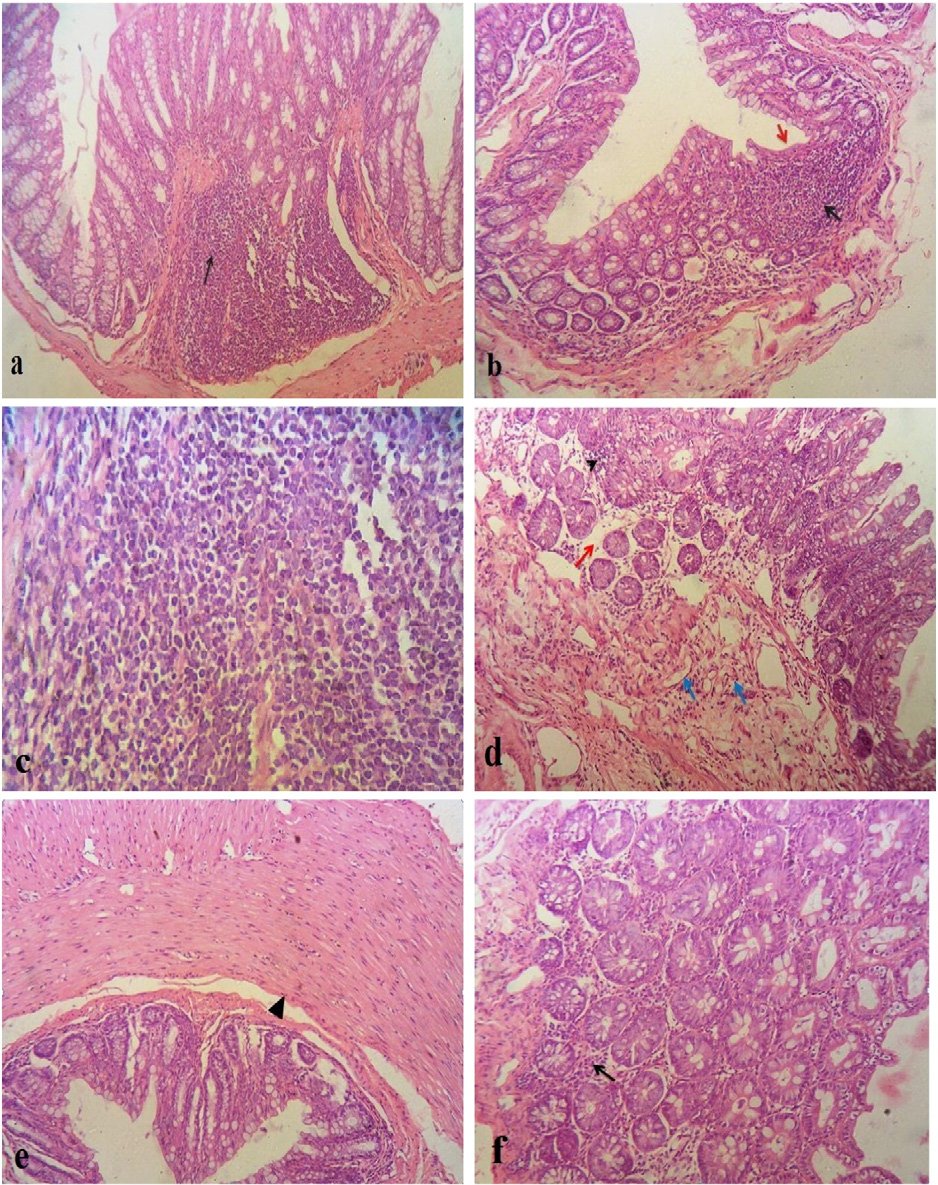
H&E sections of the large intestine of groups G3 (figures a, b, c, d and e) and G2 (figure f). a, b: marked inflammatory infiltrate of mucosa and submucosa in colonic wall (black arrow), with surface erosion (red arrow) (20 × and 10 ×, respectively), c: higher magnification of the inflammatory infiltrate showing eosinophils, lymphocytes and macrophages (40 ×), d: areas of colonic mucosa showing chronic inflammatory cell infiltrate (arrow head) within edematous stroma (red arrow) and congested blood vessels in submucosa (blue arrows) (10 ×), e: hyperplastic muscle layer (arrow head) is evident (10 ×), f: spotty infiltration by chronic inflammatory cells (black arrow) with hyperplastic colonic glands (20 ×).

Naked eye examination of large intestine showed bloated, congested large intestine with loose diarrheic stool. Histopathological examination of G3 showed marked inflammatory infiltrates of mucosa and submucosa in colonic wall. In addition, the inflammatory infiltrates were composed mainly of eosinophils, lymphocytes and macrophages. There was also hyperplasia in colonic glands, edema in colonic submucosa, congested blood vessels in submucosa and hypertrophied muscle layer was evident. The degree of inflammation ranged between 4 and 5 degrees of inflammation. On the other hand, the histopathological examination of the three infected mice in G2 showed spotty inflammatory areas and mucosal hyperplasia and was scored as 1 to 2 degree of inflammation. *D. fragilis* did not infiltrate the colonic or caecal wall (Fig. 4).

## 4 Discussion

*Dientamoeba fragilis*is a cosmopolitan occurring protozoan parasite found in human's mucosal crypts of the large intestine from caecum to rectum (Windsor et al., 2006). There is a debate about its pathogenic role, since it was considered a harmless commensal parasite for many years. Nevertheless, recent studies demonstrated that it may induce disease with diverse symptoms. Such symptoms tend to improve in most cases with the elimination of the parasite by anti-parasitic treatment (Stark et al., 2010). Other studies reported that infection with *D. fragilis* could have a self-limiting character because patients improved without treatment (Stumpel et al., 2006, Vandenberg et al., 2006).

In the same line of evidence, there is a current discussion regarding the pathogenic mechanism of *D. fragilis* and its pathogenic role. It has been reported that *D. fragilis* causes colitis by an invasive ulcerative process. This conclusion was based on the discovery of numerous ulcers in the intestine of a female patient infected with *D. fragilis* (Shein and Gelb, 1983). Furthermore, flat ulcerations together with acute and chronic inflammation, could be demonstrated in a biopsy taken from this patient. Another study showed that there were inflammatory changes in the mucosa of the rectum and sigmoid colon in *D. fragilis*-infected patients. In particular, superficial rounded infiltrations of cells in loosened stroma were observed, giving a picture of low-grade chronic proctitis (Ockert and Schulz, 1972). On the other hand, it was shown that multiplication of *D. fragilis* in the presence of suitable bacterial intestinal flora could cause disturbance of intestinal functions. It has been suggested that, the association of bacteria with *D. fragilis*, can develop amplified intestinal secretions, that may lead to the development of a suitable environment for the colonization by, and multiplication of *D. fragilis* (Lamy, 1960).The present study was performed to study the infectivity and pathogenicity of *D. fragilis* and to determine the proper number of the parasite for experimental mice infection.

In this study, all mice orally inoculated with a high dose of 4 × 10^6^ of *D. fragilis* became infected. The infected mice showed loss of weight and diarrhea. This is in agreement with a previous study showing that mice infected with *D. fragilis* experienced an average weight loss of 2.38g (12%) compared with the control group (Munasinghe et al., 2013).The presence or absence of symptoms associated with *D. fragilis* infection could be due to the presence of different genotypes of *D. fragilis*. This will be similar to what it has been described in other enteric protozoa, such as *G. lamblia* (Barratt et al., 2011). An analysis of *D. fragilis* ribosomal genes proved the existence of two genetically distinct pathogenic and nonpathogenic variants (Johnson and Clark, 2000). Nevertheless, only one *D. fragilis* genotype has been described in symptomatic and asymptomatic patients by several studies (Vandenberg et al., 2006, Johnson et al., 2004, Rayan et al., 2010). Difference in parasitic load in the colon can also play a role, observing a dose depending onset of symptoms. When the number of protozoa exceeds this threshold, their pathogenic effect can be observed. So, in the presence of appropriate intestinal bacterial flora, a gradual physiologic change of the protozoan cell could cause a qualitative alteration of the parasite's enzyme activity. Theoretically, a higher parasitic DNA load would be expected in patients with more clinical symptoms compared to asymptomatic (de Jong et al., 2014).

For a long time, it was thought that *D. fragilis* had no cyst stage and the parasite had only a trophozoite stage (Girginkardeşler et al., 2008, Ockert and Schmidt, 1975, Ghazanchaei et al., 2014). In this study, examination of stool from infected mice showed trophozoite in its rounded form, which transformed into irregular forms after minutes in the MBD culture media. In addition, it was observed that the ingested rice starch was present in its cytoplasm. Moreover, a presumptive cyst stage with its characteristic appearance was present in infected mice feces. The cyst presented a distinct thick cyst wall, with a membranous, irregular inner cyst wall located directly adjacent to the encysted parasite, surrounded by a distinctive peritrophic space. These observations were in agreement with previous findings of cysts in animal models (Munasinghe et al., 2013), as well as in human clinical samples (Stark et al., 2014).

Eida et al. reported that experimental infection of mice with *D. fragilis* resulted in histopathological changes in the caecum in the form of severe crypt hyperplasia, congestion, and edema of the lamina propria of caecal mucosa, and the interglandular epithelial cells were sloughed into the lumen. Moreover, there was penetration of multiple *D. fragilis* trophozoites in caecal glands (Eida et al., 2015). In the present study, the histopathological examination of the colon and caecum of mice in G2 showed spotty inflammation with hyperplasia in colonic glands while in heavily infected group G3 there was marked inflammatory infiltrate of mucosa and submucosa. The difference in the degree of inflammation between the two groups could be attributed to the inoculum doses used in the experiment, which means that *D. fragilis* infection is dose dependent and its pathogenic effect depends on the burden of the parasite on the large intestine, as suggested by de Jong et al. (2014). In this study, no *D. fragilis* trophozoites were detected invading the colonic mucosa as previously observed in several studies describing lesions of the large intestine in the form of colitis (Shein and Gelb, 1983), chronic or hemorrhagic proctitis (Ockert and Schulz, 1972) and appendicitis (Burrows et al., 1954, Swerdlow and Burrows, 1955), but no parasites were detected invading the underlying tissue. These pathogenic changes may be due to calprotectin release, which is a neutrophil protein, and its presence in the stool. This observation is indicative of neutrophilic infiltration into the gut lumen associated with inflammatory processes. Significantly elevated concentrations of fecal calprotectin have been demonstrated in the feces of infected mice experimentally infected with *D. fragilis* compared with the controls (Munasinghe et al., 2013). Elevated fecal calprotectin has been reported in patients suffering from intestinal disorders such as inflammatory bowel disease (IBD) and inflammatory bowel syndrome (IBS) (Costa et al., 2003). *Dientamoeba* has also been incriminated as a possible cause of IBS (Stark et al., 2010). Some parasitic infections such as giardiasis (Hanevik et al., 2007) and intestinal schistosomiasis (Bustinduy et al., 2013) showed similar elevated levels of calprotectin. Calprotectin is thought to induce inflammation through induction of pro-inflammatory chemokines, adhesion molecules and β_2_-integrin, thereby mediating leukocyte recruitment, adhesion, and trans-endothelial migration to inflamed tissue (Viemann et al., 2007, Foell et al., 2009). Moreover, the neutrophil migration and protein secretion causes epithelial damage or cell death (apoptosis), which leads to increased intestinal permeability and increased secretion of chloride ion, thus causing diarrhea in symptomatic patients (Lam et al., 2014).

In conclusion, this study revealed that large numbers of *D. fragilis* are needed for oral infection of mice. Despite its non-invasive nature, it can induce severe inflammation in the large intestinal mucosa if given in large doses. On the other hand, mice are considered a suitable animal model for *D. fragilis* experimental infection and this favors the study of the detailed biology and pathogenic mechanisms of this parasite. This is of great relevance to the advancement in the understanding of the pathology of this emerging parasite. This study suggests the presence of a cyst stage in the life cycle of *D. fragilis*. Further studies are recommended to evaluate the cyst infectivity to different lab animals and to study the ultrastructure of cysts, as well as the sequential changes of encystation through scanning and transmission EM.

## Conflict of interest statement

We declare that we have no conflict of interest.

## Sources of funding

This study received no specific grant from any funding agency in the public, commercial or not-for-profit sectors.

## References

[bb0005] Ayadi A., Bahri I. (1999). *Dientamoeba fragilis*: pathogenic flagellate?. Bull. Soc. Pathol. Exot..

[bb0010] Banik G., Barratt J., Marriott D., Harkness J., Ellis J., Stark D. (2011). A case-controlled study of *Dientamoeba fragilis* infections in children. Parasitology.

[bb0015] Barratt J.L., Harkness J., Marriott D., Ellis J.T., Stark D. (2011). A review of *Dientamoeba fragilis* carriage in humans: several reasons why this organism should be considered in the diagnosis of gastrointestinal illness. Gut Microbes.

[bb0020] Burrows R.B., Swerdlow M.A., Frost J.K., Leeper C.K. (1954). Pathology of *Dientamoeba jmgilis* infections of the appendix. Am. J. Trop. Med. Hyg..

[bb0025] Bustinduy A.L., Sousa-Figueiredo J.C., Adriko M., Betson M., Fenwick A., Kabatereine N. (2013). Fecal occult blood and fecal calprotectin as point-of-care markers of intestinal morbidity in Ugandan children with *Schistosoma mansoni* infection. Negl. Trop. Dis..

[bb0030] Camp R.R., Mattern C.F., Honigberg B. (1974). Study of *Dientamoeba fragilis* Jepps & Dobell I. Electronmicroscopic observations of the binucleate stages. II. Taxonomic position and revision of the genus. J. Protozool..

[bb0035] Clark C.G., Diamond L.S. (2002). Methods for cultivation of luminal parasitic protists of clinical importance. Clin. Microbiol. Rev..

[bb0040] Costa F., Mumolo M., Bellini M., Romano M., Ceccarelli L., Arpe P. (2003). Role of faecal calprotectin as non-invasive marker of intestinal inflammation. Dig. Liver Dis..

[bb0045] Crotti D., Annibale M., Fonzo G., Lalle M., Caccio S., Pozi E. (2005). *Dientamoeba fragilis* is more prevalent than *Giardia duodenalis* in children and adults attending a day care centre in Central Italy. Parasite.

[bb0050] Cuffari C., Oligny L., Seidman E.G. (1998). *Dientamoeba fragilis* masquerading as allergic colitis. J. Pediatr. Gastroenterol. Nutr..

[bb0055] de Jong M.J., Korterink J.J., Benninga M.A., Hilbink M., Widdershoven J., Deckers-Kocken J.M. (2014). *Dientamoeba fragilis* and chronic abdominal pain in children. A case–control study. Arch. Dis. Child..

[bb0060] Eida O.M., Eida A.M., Eida M.M., Dessouki A.A. (2015). The effect of *Nigella sativa* aqueous extract on *Dientamoeba fragilis*: an in vivo experimental study. PUJ.

[bb0065] Foell D., Wittkowski H., Roth J. (2009). Monitoring disease activity by stool analyses: from occult blood to molecular markers of intestinal inflammation and damage. Gut.

[bb0070] Garcia L.S. (2009). Practical Guide to Diagnostic Parasitology.

[bb0075] Ghazanchaei A., Shargh S., Shabani M., Najafi M., Nourazaria S. (2014). Detection of *Dientamoeba fragilis* in patients referred to Chaloos medical care centers by nested-polymerase chain reaction (PCR) method. Afr. J. Biotechnol..

[bb0080] Girginkardeşler N., Kurt Ö., Kilimcioğlu A.A., Ok Ü.Z. (2008). Transmission of *Dientamoeba fragilis*: evaluation of the role of *Enterobius vermicularis*. Parasitol. Int..

[bb0085] Gray T.J., Kwan Y.L., Phan T., Robertson G., Cheong E.Y., Gottlieb T. (2013). *Dientamoeba fragilis*: a family cluster of disease associated with marked peripheral eosinophilia. Clin. Infect. Dis..

[bb0090] Hamano S., Asgharpour A., Stroup S.E., Wynn T.A., Leiter E.H., Houpt E. (2006). Resistance of C57BL/6 mice to amoebiasis is mediated by nonhemopoietic cells but requires hemopoietic IL-10 production. J. Immunol..

[bb0095] Hanevik K., Hausken T., Morken M.H., Strand E.A., Mørch K., Coll P. (2007). Persisting symptoms and duodenal inflammation related to *Giardia duodenalis* infection. J. Infect..

[bb0100] Jepps M.W., Dobell C. (1918). *Dientamoeba fragilis* ng, n. sp., a new intestinal amoeba from man. Parasitology.

[bb0105] Johnson J.A., Clark C.G. (2000). Cryptic genetic diversity *Indientamoeba fragilis*. J. Clin. Microbiol..

[bb0110] Johnson E.H., Windsor J.J., Clar C.G. (2004). Emerging from obscurity: biological, clinical, and diagnostic aspects of *Dientamoeba fragilis*. Clin. Microbiol. Rev..

[bb0115] Lam Y.A., Warouw S.M., Wahani A.M., Manoppo J.I., Salendu P.M. (2014). Correlation between gut pathogens and fecal calprotectin levels in young children with acute diarrhea. Paediatr. Indones..

[bb0120] Lamy L. (1960). *Dientamoeba fragilis* recherche, culture, frequency, interet et caracteres pathoges. Bull. Soc. Pathol. Exot. Fil..

[bb0125] Levine N., Corliss J., Cox F., Deroux G., Grain J., Honigberg B. (1980). A newly revised classification of the protozoa. J. Protozool..

[bb0130] LiPuma J., Currie B., Lum G., Vandamme P. (2007). *Burkholderia*, *Stenotrophomonas*, *Ralstonia*, *Cupriavidus*, *Pandoraea*, *Brevundimonas*, *Comamonas*, *Delftia*, and *Acidovorax*. Manual Clin. Microbiol..

[bb0135] Munasinghe V.S., Stark D., Ellis J. (2012). New advances in the in-vitro culture of *Dientamoeba fragilis*. Parasitology.

[bb0140] Munasinghe V.S., Vella N.G., Ellis J.T., Windsor P.A., Stark D. (2013). Cyst formation and faecal–oral transmission of *Dientamoeba fragilis* — the missing link in the life cycle of an emerging pathogen. Int. J. Parasitol..

[bb0145] Norberg A., Nord C., Evengård B. (2003). *Dientamoeba fragilis*—a protozoal infection which may cause severe bowel distress. Clin. Microbiol. Infect..

[bb0150] Ockert G., Schmidt T. (1975). On the epidemiology of *Dientamoeba fragilis* Jepps and Dobell 1918. 4th communication evidence of *Dientamoeba fragilis* in *Enterobius* eggs using isoelectric point determination. J. Hyg. Epidemiol. Microbiol. Immunol..

[bb0155] Ockert G., Schulz U. (1972). Pathogenetic role of *Dientamoeba fragilis*. Dtsch. Gesundheitsw..

[bb0160] Rayan H., Ismail O., El Gayar E. (2010). Molecular diagnosis and genotyping of *Dientamoeba fragilis* by polyerase chain reaction compared to microscopy and culture. PUJ.

[bb0165] Sawangjaroen N., Luke R., Prociv P. (1993). Diagnosis by faecal culture of *Dientamoeba fragilis* infections in Australian patients with diarrhoea. Trans. R. Soc. Trop. Med. Hyg..

[bb0170] Shein R., Gelb A. (1983). Colitis due to *Dientamoeba fragilis*. Am. J. Gastroenterol..

[bb0175] Stark D., Beebe N., Marriott D., Ellis J., Harkness J. (2005). Prospective study of the prevalence, genotyping, and clinical relevance of *Dientamoeba fragilis* infections in an Australian population. J. Clin. Microbiol..

[bb0180] Stark D.J., Beebe N., Marriott D., Ellis J.T., Harkness J. (2006). Dientamoebiasis: clinical importance and recent advances. Trends Parasitol..

[bb0185] Stark D., Barratt J., Roberts T., Marriott D., Harkness J., Ellis J.A. (2010). Review of the clinical presentation of dientamoebiasis. Am. J. Trop. Med. Hyg..

[bb0190] Stark D., Garcia L., Barratt J., Phillips O., Roberts T., Marriott D. (2014). Description of *Dientamoeba fragilis* cyst and precystic forms from human samples. J. Clin. Microbiol..

[bb0195] Stumpel O., Tolboom J., Warris A. (2006). *Dientamoeba fragilis*, vooral bij kinderen pathogeen. Tijdschr. Infectieziekten.

[bb0200] Swerdlow M.A., Burrows R.B. (1955). *Dientamoeba fragilis*, an intestinal pathogen. J. Am. Med. Assoc..

[bb0205] Vandenberg O., Peek R., Souayah H., Dediste A., Buset M., Scheen R. (2006). Clinical and microbiological features of dientamoebiasis in patients suspected of suffering from a parasitic gastrointestinal illness: a comparison of *Dientamoeba fragilis* and *Giardia lamblia* infections. Int. J. Infect. Dis..

[bb0210] Viemann D., Barczyk K., Vogl T. (2007). MRP8/MRP14 impairs endothelial integrity and induces a caspase-dependent and-independent cell death program. Blood.

[bb0215] Windsor J.J., Macfarlane L., Clark C.G. (2006). Internal transcribed spacer dimorphism and diversity in *Dientamoeba fragilis*. J. Eukaryot. Microbiol..

